# HIF and MYC signaling in adrenal neoplasms of the neural crest: implications for pediatrics

**DOI:** 10.3389/fendo.2023.1022192

**Published:** 2023-06-08

**Authors:** Nicole Bechmann, Frank Westermann, Graeme Eisenhofer

**Affiliations:** ^1^ Institute of Clinical Chemistry and Laboratory Medicine, University Hospital Carl Gustav Carus, Medical Faculty Carl Gustav Carus, Technische Universität Dresden, Dresden, Germany; ^2^ Hopp Children’s Cancer Center Heidelberg (KiTZ), Heidelberg, Germany; ^3^ Division of Neuroblastoma Genomics, German Cancer Research Center (DKFZ), Heidelberg, Germany; ^4^ Department of Medicine III, University Hospital Carl Gustav Carus, Medical Faculty Carl Gustav Carus, Technische Universität Dresden, Dresden, Germany

**Keywords:** pheochromocytoma, neuroblastoma, catecholamines, sympathoadrenal cell lineage, hypoxia, MYC, neural crest, paraganglioma

## Abstract

Pediatric neural crest-derived adrenal neoplasms include neuroblastoma and pheochromocytoma. Both entities are associated with a high degree of clinical heterogeneity, varying from spontaneous regression to malignant disease with poor outcome. Increased expression and stabilization of HIF2α appears to contribute to a more aggressive and undifferentiated phenotype in both adrenal neoplasms, whereas *MYCN* amplification is a valuable prognostic marker in neuroblastoma. The present review focuses on HIF- and MYC signaling in both neoplasms and discusses the interaction of associated pathways during neural crest and adrenal development as well as potential consequences on tumorigenesis. Emerging single-cell methods together with epigenetic and transcriptomic analyses provide further insights into the importance of a tight regulation of HIF and MYC signaling pathways during adrenal development and tumorigenesis. In this context, increased attention to HIF-MYC/MAX interactions may also provide new therapeutic options for these pediatric adrenal neoplasms.

## 1 Introduction

The neural crest comprises a multipotent stem cell population that gives rise to a large variety of cell types, including sympathetic and parasympathetic ganglia and chromaffin cells. From these cell types neuroblastomas or pheochromocytomas/paragangliomas can originate. Both tumor entities can occur in the adrenal gland in some cases already soon after birth or in early childhood and are characterized by clinical and molecular heterogeneity. In both neural crest-derived neoplasms, it is discussed that increased expression and stabilization of HIF2α contributes to a more aggressive and undifferentiated phenotype (1–4). However, dependent on the context HIF2α may also promote tumor suppressive activities, which was reported for both neuroblastoma (5–7) and non-neural crest-derived neoplasms (8–10). The occurrence of *MYCN* amplification in approximately 20% of neuroblastomas, which correlates with high-risk disease and poor prognosis (11), further underscores the importance of MYC and HIF signaling pathways in these adrenal neoplasms derived from the neural crest.

**Figure 1:**
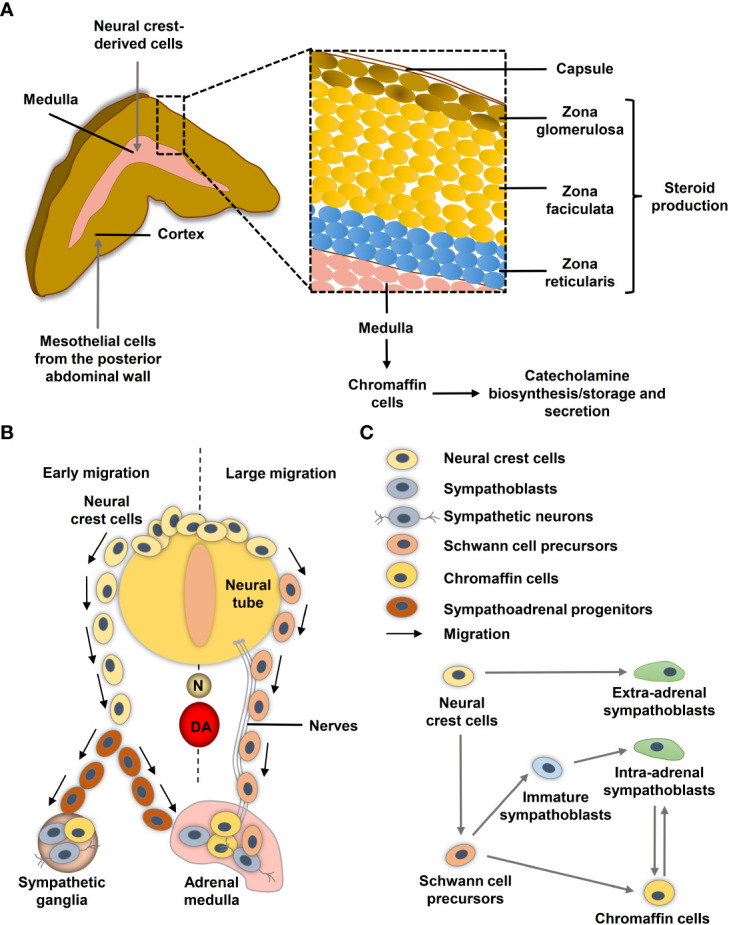
Neural crest and adrenal chromaffin cell development. **(A)** Human adult adrenal zonation. **(B)** Schematic illustration of human adrenal and sympathetic ganglia development from the neural crest. **(C)** Chromaffin cell and sympathoblast development from neural crest-derived cells in humans (adapted from (12)). DA, dorsal aorta; N, notochord.

The human adrenal gland composes two distinct tissues, the outer cortex and the inner medulla under a common capsule (**Figure 1A**). The steroid hormone producing adrenal cortex (mineralocorticoids, glucocorticoids and androgens) arises from the coelomic mesoderm of the urogenital ridge, while the adrenal medulla arises from the neural crest and secretes the catecholamines epinephrine and less prominently norepinephrine (13, 14). During neurulation, the neural folds arise at the boundary between non-neural and neural ectoderm and subsequently coalesce in the midline to form the neural tube. The pre-migrating neural crest cells localized at the neural fold are characterized by their epithelial phenotype associated with strong cell-cell junctions. To initiate migration, neural crest cells must undergo an epithelial-mesenchymal transition (EMT) to acquire a motile phenotype and lose their cell-cell junction (15).

During migration, neural crest cells proliferate extensively to generate enough precursor cells to colonize their targets. Ventrally migrating neural crest cells colonize the paravertebral sympathetic ganglia in the trunk and the chromaffin cells of the adrenal medulla and paraganglia (16) (**Figure 1B**). After reaching their targets, neural crest-derived cells differentiate into the appropriate cellular subtypes. Migration and differentiation of the distinct neural crest cell derivatives is orchestrated by expression of lineage specific markers that are influenced by signals of the microenvironment such as hypoxia.

Single-cell RNA sequencing (scRNA-seq) revealed differences in the transitions between sympathoadrenal fates in humans and mice (12). In human embryos, Schwann cell precursors (SCPs; *SOX10* ^+^) derived from neural crest cells connect to *STMN2^+^ISL1^+^PRPH^−^* sympathoblasts and the *CHGA^+^PENK^+^PNMT^+^* chromaffin cells through a ‘fork-like’ transition (**Figure 1C**) (12). Cells within this first transition show overlaps between both expression programs in form of *SOX10* ^+^/*ISL1* ^+^/*HAND2* ^+^, *SOX10* ^+^/*ISL1* ^+^ and *SOX10* ^+^/*HAND2* ^+^ transitory cells (12). A second transition between sympathoblasts and chromaffin cells (markers: *SOX4*, *BEX1*, *RAMP1*, *PENK*) was identified (**Figure 1C**). In mice, chromaffin cells of the adrenal medulla arise from an intermediate bridge cell population (transition 1) and can transit to sympathoblasts (12, 17). While cellular properties, such as high proliferative capacity and motility, are crucial during embryogenesis, they may become problematic later in life when the same properties contribute to tumor aggressiveness and metastasis. Low-risk neuroblastomas share similarities to differentiated late sympathoblasts (also named neuroblasts in the context of neuroblastomas), whereas high-risk *MYCN*-amplified neuroblastomas resemble more undifferentiated sympathoblasts (18–20). Bedoya-Reina et al. further showed that tumor cells enriched in high-risk neuroblastomas resemble a subtype of TRKB+ cholinergic progenitor population that they previously identified in human postnatal adrenal glands (20). An undifferentiated phenotype is also associated with a higher risk of metastasis in pheochromocytomas and extra-adrenal paragangliomas (21, 22), although the exact cellular origin of the various subgroups is still unclear.

In the present review, we describe current knowledge about the interplay between MYC and HIF signaling during neural crest, adrenal development and the potential role of these signaling pathways in catecholamine-producing neural crest-derived neoplasms.

## 2 Interplay of MYC and HIF signaling

**Figure 2:**
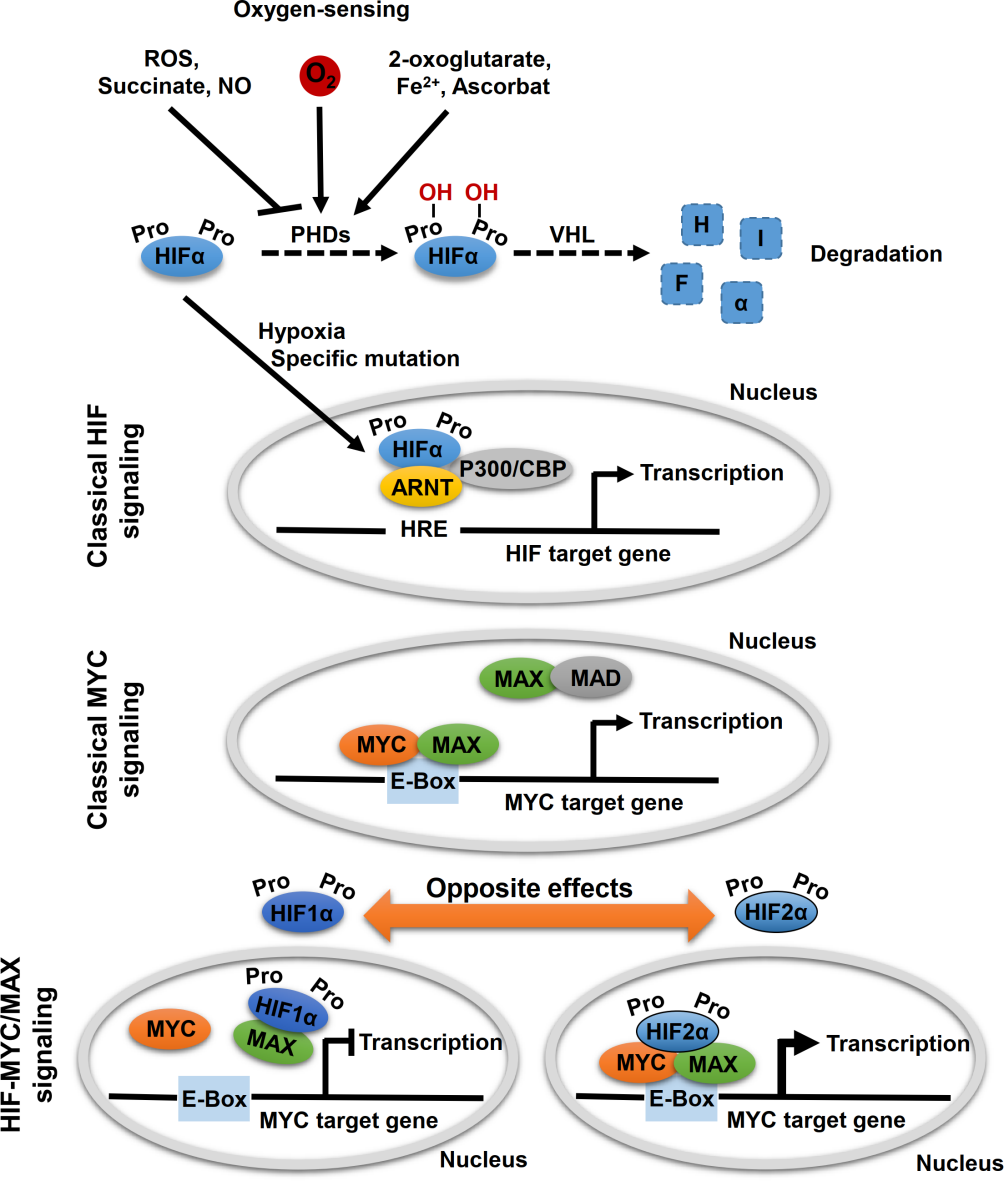
Oxygen-sensing and HIF-MYC signaling. Key molecule of oxygen-sensing system are hypoxia-inducible factors (HIFs), which regulate the transcription of a wide range of oxygen responsive genes. In presence of oxygen, HIF proline hydroxylases (PHDs) hydroxylate proline residues within HIFα subunits, which lead to proteasomal degradation of HIFα by the von Hippel-Lindau (VHL) tumor suppressor. Hypoxic conditions (absence of oxygen) or specific mutations, that affect HIFα degradation, lead to a stabilization of HIFα. Subsequently, HIFα subunits translocated to the nucleus where they form a complex with aryl hydrocarbon receptor nuclear translocator (ARNT, also known as HIFβ) and specific co-factors and bind to hypoxia-responsive elements (HREs) leading to transcription of HIF target genes (classical HIF signaling). MYC proteins encoded by MYC proto-oncogenes (*c-MYC, MYCN, l-MYC*) are localized in the nucleus and form heterodimers with myc-associated factor X (MAX), which enables recognition by the hexameric DNA sequence CACGTG (E-Box) and subsequent transcriptional activation of MYC target genes (classical MYC signaling). Moreover, other binding partners of MYC and MAX also regulate MYC target gene transcription, including MAX dimerization protein 1 (MAD) and HIFαs. Thereby, opposite effects on MYC target gene expression were described for HIF1α and HIF2α. While HIF1α dimerizes with MAX and thereby suppresses binding to the E-box, HIF2α leads to stabilization of the MYC/MAX complex and thus to activation of MYC target genes.

MYC proteins are key regulators of cell fate and part of a network of interacting transcription factors, that regulate expression of various genes involved in, for example, cell proliferation, differentiation and metabolism (23). In addition to c-MYC, the MYC protein family also includes MYCN and l-MYC. These helix–loop–helix leucine zipper transcription factors heterodimerize with MYC-associated protein X (MAX) (**Figure 2**). After heterodimerization, MYC/MAX binds to specific DNA sequence (CANNTG) called E-box to activate or repress transcription of more than 15% of all genes in cells (24–26). MAX itself is under tight control by a network of protein-protein interactions with MAX dimerization protein (MXD1, also known as MAD) and MAX interactor 1 (MXI1, also known as MAD2) (27). Other factors, such as the hypoxia inducible factor (HIF) 1α and 2α, further affect binding of MYC/MAX to the E-box.

HIF proteins are the main regulators of oxygen sensing in cells (**Figure 2**). Under normoxic conditions (presence of oxygen), proline residues of the HIFα subunits are hydroxylated by oxygen- and α-ketoglutarate-dependent prolyl hydroxylases (PHDs). This allows for recognition of the HIFα subunits by the von Hippel–Lindau protein (VHL) and their subsequent degradation by proteasomes (**Figure 2**). Under hypoxic conditions (absence of oxygen), HIFα subunits are stabilized and form a complex with aryl hydrocarbon receptor nuclear translocator (ARNT, also known as HIFβ) and several co-factors, including CREB-binding protein and p300. The HIF complex binds to hypoxia-response element (HRE) of the gene promoter for transactivation, thereby regulating genes involved in angiogenesis, pH regulation, glycolysis, and glucose transport, which enable cellular adaptation to hypoxia.

The two main HIFα subunits, HIF1α and HIF2α, have mostly complementary functions, but their activity differs temporally: while HIF1α predominantly mediates the acute response to severe hypoxia, HIF2α modulates adaption to chronic or even mild hypoxia (4). In addition, HIF1α is ubiquitously expressed, while HIF2α has a more restricted expression profile (28). Both HIFαs differ in their targets; with HIF1α specifically activates genes involved in glycolysis and HIF2α preferentially activates VEGF, transforming growth factor-α (TGFα), lysyl oxidase (Lox), Oct4 and Cyclin D1 (29, 30). Evidence suggests that HIF2α preferentially promotes tumorigenesis and affects the differentiation status of different tumor entities (29). The effects on the tumor phenotype might be explained by the HIF2α-induced expression of the stem cell factor Oct4 and the transcriptional activation of c-Myc (29, 30). For example, in hepatocellular carcinoma (HCC) tumor tissue, c-Myc expression showed a positive correlation with HIF2α but not with HIF1α (31). Knockdown of HIF2α diminished expression of c-Myc in HCC cells, suppressed hypoxia-related proliferation, and induced apoptosis (31). On the other hand, Yang et al. revealed that HCC patients with high HIF2α protein levels had longer overall survival indicating a tumor suppressor function of HIF2α in these tumors (10). In colorectal cancer cell lines, HIF2α regulated expression of *c-Myc* under chronic hypoxia and thereby controls sensitivity to 5-flurouracil (32). HIF1α and HIF2α play distinct roles in colon cancer (9). It was also shown that *EPAS1* was significantly reduced in primary adenocarcinoma samples of the colon compared to histopathologically non-neoplastic tissue, while no difference was found for *HIF1α* (33). Renal cell carcinoma cells expressing almost exclusively HIF2α exhibit lower genomic instability, which correlates with enhanced c-MYC-dependent expression of genes involved in DNA repair (34). Moreover, emerging evidence suggests that MYC regulates the levels and activity of HIF1α (35–38).

In addition, other HIF-dependent mechanisms modulating MYC/MAX complex formation and promoter occupancy have also been proposed, with HIF1α and HIF2α also exhibiting opposing effects (**Figure 2**) (26, 39, 40). HIF1α antagonized c-MYC activity by displacing c-MYC from transcription factor Sp1 binding that is required for MYC promotor activation, while phosphorylation of HIF2α prevents HIF2α from competing with MYC for Sp1, thereby enhancing Sp1-c-Myc activity (41–44). Under chronic hypoxia, HIF1α promotes c-MYC degradation and induces expression of MAX interactor 1 (MXI1) that furthermore inhibits MYC target gene expression (45, 46). HIF1α can further bind to MAX to thereby disrupt the formation of the MYC/MAX complex (39). On the other hand, HIF2α promotes MYC activity by enhancing Sp1-c-Myc activity as mentioned above. HIF2α enhances c-MYC activity by stabilizing the MYC/MAX complex (**Figure 2**) (45, 47). This effect appears to be much stronger than HIF1α-mediated degradation of c-MYC in tumor cells, leading to activation of MYC under hypoxic conditions (47). Moreover, HIF has been shown to promote proteosomal degradation of MYC under chronic hypoxic conditions in dependence of the used cell system (45, 48–50).

Most of the aforementioned studies addressing the interaction between HIF and MYC focused exclusively on the effect of c-Myc. Whether the mechanisms are also applicable to MYCN remains unclear. Due to the tissue- and cell-specific differences in c-MYC and MYCN expression and the resulting different roles (e.g. during neural crest development and tumorigenesis), it can be assumed that the interaction with HIF1α and HIF2α is also different.

## 3 HIF signaling during neural crest and adrenal development

The ability to sense oxygen (**Figure 2**) is crucial for the survival of water- and air-breathing organisms. Adrenomedullary chromaffin cells as well as glomus cells of the carotid body that both arise during neural crest development synthesize and release catecholamines in response to hypoxic stress (51, 52). Chemoreceptors in the carotid body are relatively non-sensitive to hypoxia in the neonatal period and achieve adult-like sensitivity by 2-3 weeks postnatally in rats. In contrast, rat adrenomedullary chromaffin cells are most sensitive to hypoxia in the perinatal period, and this sensitivity is gradually lost until it is largely gone by 2-3 weeks postnatally (53–55). The oxygen sensitivity of these neural crest-derived cells suggests involvement of the HIF signaling pathways already during embryogenic development.

Under physiological conditions, hypoxia occurs in amniotic embryos prior to the onset of a functional blood circulation (56). In rodents and chicken embryos, low oxygen concentrations (5%) have been shown to be required until the neural tube has closed and the cells of the cranial neural crest have emigrated (57, 58). Studies in Xenopus, chicken, and quail embryos indicate that HIF1α and ARNT are expressed together and ubiquitously in the developing embryo (up to HH14; HH stage in chicken embryo), whereas HIF2α has a more pronounced expression pattern that includes tissues that do not express HIF1α (58, 59). Trunk neural crest cells that give rise to the adrenal medulla and the sympathetic ganglia form mainly after vascularization and are not affected by high oxygen or deletion of HIF1α (57, 58, 60). However, stabilization of HIFα in neural crest cells seems to be controlled by oxygen-dependent (hypoxia) and oxygen-independent mechanisms (pseudohypoxia) (61), similar to what is already known for neuroblastoma, pheochromocytoma and paraganglioma (PPGL) (62, 63).

**Figure 3:**
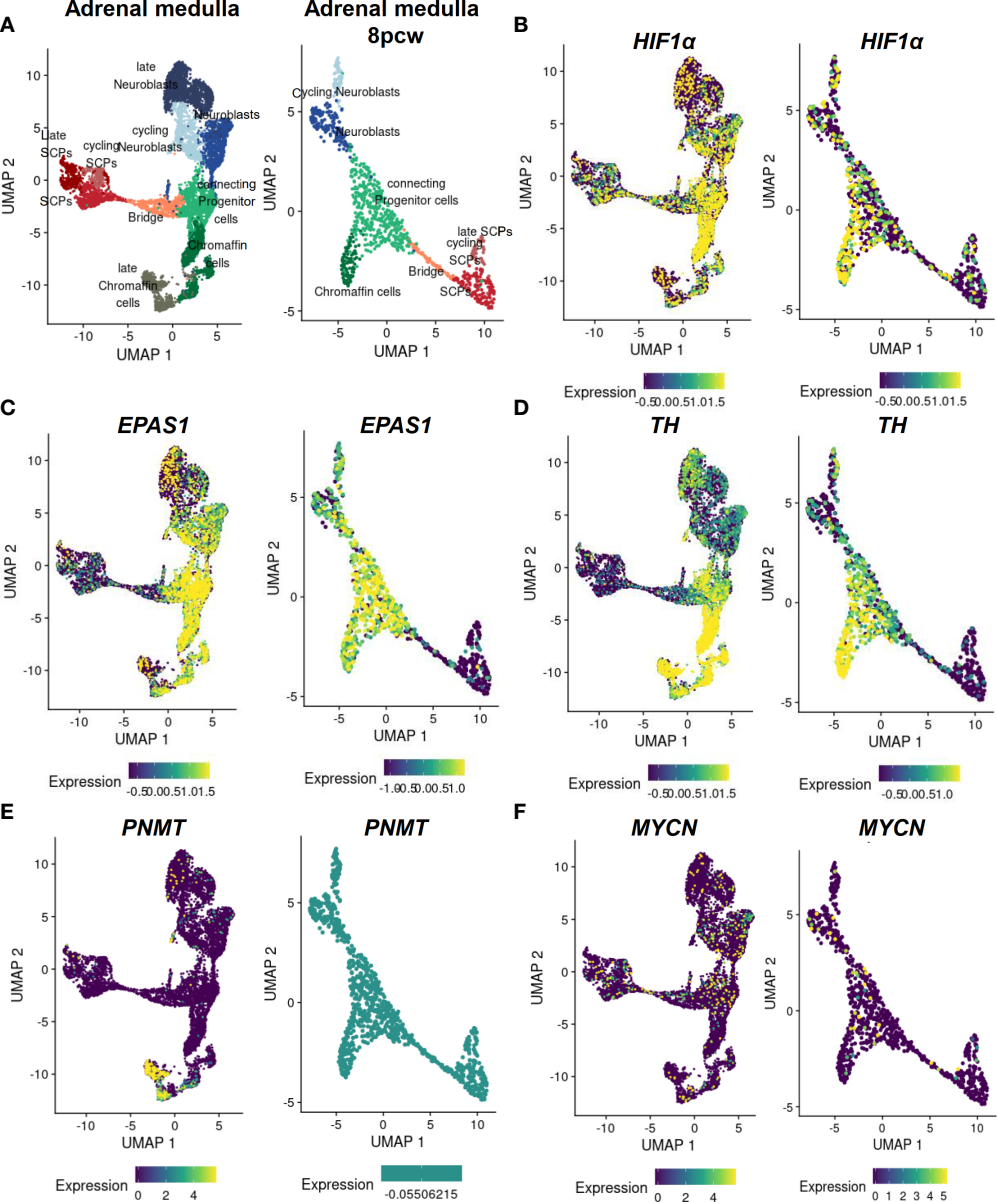
Expression of *MYCN*, *HIF*s and chromaffin cell markers in the developing human adrenal medulla. Expression patterns of genes of interest obtained by single nucleus RNA sequencing data (18) of the developing human adrenal medulla were visualized by https://adrenal.kitz-heidelberg.de/developmental_programs_NB_viz/ (last request: March 2022). **(A)** UMAP plot of adrenal medullary cells of developing adrenal and of the fetal adrenal eight weeks post conception (8pcw). Different colors highlight different cell populations. Expression pattern of **(B)** *HIF1α*, **(C)** *EPAS1*, **(D)** *TH*, **(E)** *PNMT* and **(F)** *MYCN* in the developing human adrenal and of the fetal adrenal eight weeks post conception. The color indicates the normalized gene expression (blue low expression; yellow high expression). Data on *c-Myc* and *MAX* expression were not available. UMAP: Uniform manifold approximation and projection; SCP, Schwann cell precursors.

Inhibition of Hif1α in Xenopus laevis and zebrafish embryos results in complete blockade of neural crest migration by controlling chemotaxis and epithelial-mesenchymal transition (61). In mice selectively deficient in HIF1α in ISL^+^ cells, the development of sympathetic ganglia and chromaffin cells is impaired (64). Mice with deletion in Vhl restricted to tyrosine hydroxylase (Th)-positive cells exhibit atrophy of the carotid body, adrenal medulla and sympathetic ganglia; this was associated a striking intolerance to systemic hypoxia that could lead to death (65). *HIF1α* is diffusely expressed in all cell types of the developing human adrenal medulla with a stronger accumulation in the connecting progenitor cells and early chromaffin cells (**Figures 3A, B**), while expression of *HIF2α (gene also known as EPAS1)* appears to be restricted to specific cell populations (**Figure 3C**). snRNA-seq revealed a high expression of *HIF2α* in early chromaffin cells and neuroblasts as well as in the connecting progenitor cells, whereas SCPs and late neuroblasts as well as late chromaffin cells showed only limited expression of *HIF2α* in isolated cells (**Figures 3C, D**) (18).

During neural crest development, HIF2α is expressed in migrating trunk neural crest cells and sympathetic neuroblasts in human, murine, and avian embryos (66). In the sympathetic ganglia of human embryos, HIF2α is expressed at embryonic week 6.5, but expression is lost at later stages (67). *HIF2α* knockout mice exhibit severe sympathetic nervous system (SNS) abnormalities associated with mid-gestation lethality (68). Both overexpression and silencing of HIF2α *in vivo* delay neural crest development, induce proliferation and self-renewal capacity of neural crest cells, and reduce the proportion of neural crest cells that migrate ventrally to sympathoadrenal sites, suggesting that HIF2α needs to be tightly controlled during normal development of the trunk neural crest (66). These data underline the importance of HIF2α in chromaffin cell and sympathoblast differentiation, which may further provide a rational for the various differentiation states of adrenal neoplasms.

In mice, inactivation of Phd3 (responsible for the hydroxylation of the HIFαs that initiates their proteasomal degradation) promotes survival of sympathoadrenal neurons, which appear hypofunctional despite increased numbers of Th-positive cells in the adrenal medulla and carotid body, due at least in part to upregulation of Hif2α but not Hif1α (69). Moreover, hypoxia induced catecholamine release by chromaffin cells, and here in particular regulated by HIF2α (68, 70), is crucial to maintain physiological homeostasis of the fetus before sympathetic innervation is fully complete (71, 72). During birth, increased catecholamine release facilitates adequate hemodynamic adjustment and stimulates surfactant production by the lungs (73, 74). A deficiency of *Phd2* in the adrenal medulla of mice results in a Hif2α-mediated reduction in phenylethanolamine *N*-methyltransferase (Pnmt) associated with a reduced epinephrine biosynthesis (75). This is also consistent with studies in *Hif2α* ^-/-^ mice, which showed reduced epinephrine levels in the adrenal gland (68, 76). In the developing human adrenal medulla, expression of *PNMT* is restricted to late chromaffin cells, which do not express *HIF2α* or express *HIF2α* in a restricted manner (**Figure 3E**) (18). Reduced oxygen promotes survival and catecholaminergic differentiation, characterized by the expression of tyrosine hydroxylase, which catalyzed conversion of tyrosine to L-DOPA (precursor catecholamine biosynthesis), in neural crest stem cells and central nervous system progenitors (77, 78). It is therefore possible that hypoxia mediates stem cell function by affecting Oct4, which is controlled by HIF2α as discussed in the previous section (30).

## 4 MYC signaling during neural crest and adrenal development

Mice studies revealed that c*-Myc* and *Mycn*, but not *l-Myc*, are fundamental for normal development since targeted deletions are lethal to the embryo at mid-gestation (79–81). Tissue- and cell-specific expression patterns of Mycn and c-Myc have been observed during embryogenesis. While Mycn expression is restricted to specific cell types of epithelial tissues, including those of the developing nervous system and organs characterized by epithelial-mesenchymal interaction, c-Myc expression is restricted to mesenchymal compartments (82). A time-dependent expression of c-Myc and Mycn in the developing neural crest and neural crest cells is observed in chicken embryos (83). In the neural plate during gastrulation throughout the anterior-to-posterior axis Mycn, but not c-Myc is expressed. Neither c-Myc nor Mycn is expressed in the neural plate boarder at stage HH4-7, which later form the neural crest. When the neural tube closure starts (HH8) expression of Mycn is restricted to the ventral parts, which form the central nervous system (CNS). The expression of c-Myc begins at later stage HH8 in the dorsal neural tube later forming the neural crest. In stage HH9, c-Myc expression remains restricted to the dorsal neural crest area, while Mycn is expressed in the remaining neural tube except in the dorsum (83).

In Xenopus, c-Myc is an essential early regulator of neural crest cell formation, with expression of c-Myc localized to the neural plate boundary prior to expression of early neural crest markers (84). It has been suggested that c-Myc prevents cell fate decisions during neural crest formation, possibly via the Myc target gene *Id3* (85). Knockdown of *Id3* leads to a lack of neural crest formation in Xenopus embryos, since Id3 maintains the precursor state of neural crest cells (85). Loss of c-Myc results in a drastic reduction in the number of emigrating cells of the neural crest, due to a reduced capacity for self-renewal, increased cell death, and a shorter duration of the emigration process in chicken embryos. In this regard, c-Myc appears to bind to Miz1 rather than to the E-box to activate the cell cycle (86).

In chicken and mouse embryos, Mycn is expressed in early neural crest lineage, the central nervous system, a subset of mesoderm derivatives and endodermal epithelia, in particular (87). Deficiency of Mycn in mouse embryos leads to a reduction in mature neurons of the dorsal root ganglia and sympathetic ganglia, underscoring the importance of Mycn in the development of neurons from the neural crest (81). At early stages, Mycn appears to be involved in the proliferation of progenitor populations rather than in their differentiation per se (79). Later during neural development, Mycn has been linked to the maintenance of neural fate as it is expressed by slowly proliferating neural stem cells and is involved in the expansion and differentiation of neural progenitor cells in the CNS (81, 88, 89). In addition, Mycn promotes neural fate and differentiation in the peripheral nervous system (79, 87). After neural crest EMT, Mycn is only expressed at low levels in migrating neural crest cells followed by a further downregulation before the cells aggregate to form the ganglia (90). Mycn is expressed in regions of the neural plate destined to form the central nervous system, but not in the neighboring neural crest stem cell domain (83). Mycn expression in the neural crest domain biases cells toward a more CNS neural stem cell-like fate (expression SOX2), leading to improperly specified neural crest cells; this may play a role of priming in neuroblastoma development (83). However, overexpression of Mycn in mouse sympathoadrenal progenitors is insufficient for tumor formation in nude mice but leads to enhanced neural differentiation (91). Increased expression of Mycn in mouse neural crest cells results in neuroblastoma-like tumors, suggesting that premature exposure of neural crest cells to high Mycn levels may be important in the development of neuroblastoma (92). Our scRNA-seq data reveal an expression of *MYCN* only in single cells of the human developing adrenal medulla not restricted to a specific cell type (**Figure 3F**).

A possible time-dependent co-expression of HIFs and MYCs during neural crest and adrenal development may indicate a possible interaction of the two signaling pathways during these embryological processes, but precise data are unfortunately not available in this regard. The role of the cell-specific expression of the respective isoforms of HIF and MYC and how exactly they are related to each other remains unclear. Furthermore, most studies in this context rely on Xenopus, mouse and chicken embryo studies. Transferability to humans needs to be clarified. Nevertheless, data indicate that the embryogenic origin of the cells and the associated differentiation status influenced by HIF and MYC may contribute decisively to the phenotype of adrenal neoplasms.

## 5 HIF and MYC signaling in catecholamine-producing neoplasms of the neural crest: neuroblastoma, pheochromocytoma and paraganglioma

Pediatric neuroblastomas and PPGLs are characterized by a high degree of heterogeneity in disease presentation associated with distinct differentiation stages, affected by HIF and MYC signaling pathways.

**Figure 4:**
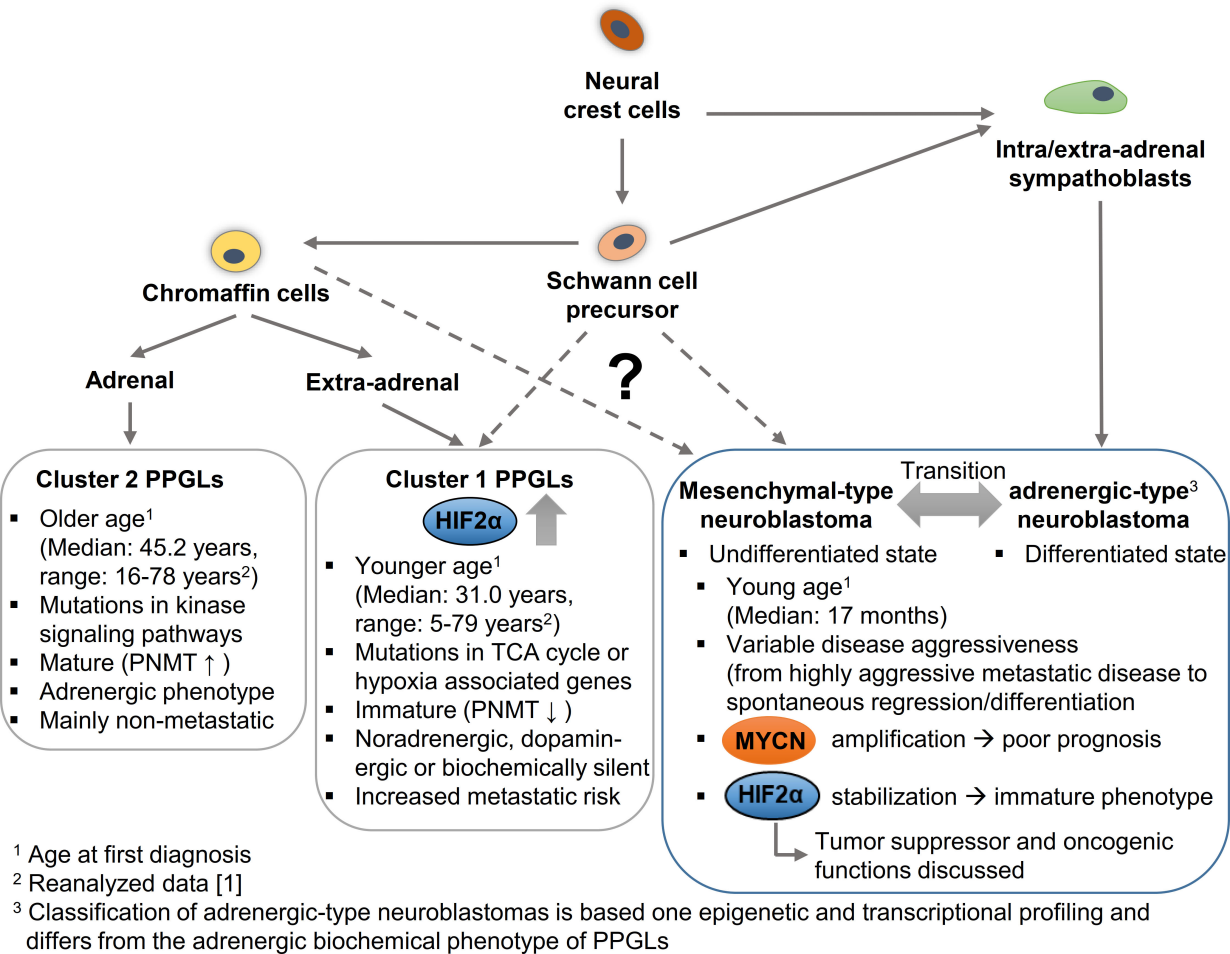
Hypothetical model to explain the cellular origin leading to different phenotypic features in pheochromocytomas/paragangliomas (PPGLs) and neuroblastomas in dependence of HIF2α and MYCN. PPGLs and neuroblastomas originate from neural crest cells; while mature neuroblastomas arise from a sympathoblast lineage, mature cluster 2 PPGLs originate form a chromaffin cell lineage. Both lineages arise via Schwann cell precursors that may give rise to more immature cluster 1 PPGLs and mesenchymal-type neuroblastomas. Increased expression and stabilization of HIF2α is associated with an immature phenotype and enhanced aggressiveness in PPGLs, while for HIF2α in neuroblastoma both an oncogenic and a tumor suppressor function are discussed. MYCN amplification correlates with a worse prognosis in neuroblastomas. PNMT, Phenylethanolamine N-methyltransferase; TCA cycle, Tricarboxylic acid cycle.

Neuroblastoma is the most common solid neoplasm in childhood and its clinical course varies from spontaneous tumor regression to fatal malignant progression (**Figure 4**); even differentiation into benign forms (ganglioneuroma or ganglionneuroblastoma) are described (93). Malignant expansion in neuroblastoma appears manly triggered by *MYCN* amplification or/and *ALK* and *ATRX* mutations (94). Additional factors that influence prognosis include the location of the primary tumor and the age of the patient. For example, adrenal neuroblastomas are more frequently *MYCN*-amplified and are often in tumor stage 4 (International Neuroblastoma Staging System Committee (INSS) system), in which the primary tumor has spread to distant organs, compared with extra-adrenal neuroblastomas (95). Children with stage 4 neuroblastoma who are younger than 1 year have a significantly better prognosis than older children at stage 4 (96).

Epigenetic and transcriptional profiling in neuroblastoma cell lines identified two distinct neuroblastoma cell identities: the undifferentiated mesenchymal-type (MES) and adrenergic-type (ADRN; to be strictly distinguished from an adrenergic phenotype in PPGLs) that can interconvert and reflect cells from different stages of differentiation (**Figure 4**) (97). Patient samples allowed a further subclassification of the adrenergic group linked to distinct clinical outcomes into *MYCN*-amplified, *MYCN* non-amplified high-risk and *MYCN* non-amplified low-risk signatures (98). High MYCN/c-MYC target gene expression is a hallmark of malignant neuroblastoma progression (99). Elevated expression of a subset of MYCN/c-MYC target genes identifies a patient subtype with poor overall survival, independent of established risk markers such as *MYCN* amplification, disease stage, and age at diagnosis (99). Malignant progression, therapy resistance and disease relapse in neuroblastoma are associated with an undifferentiated phenotype, which is linked to the broad activation/repression of MYCN/c-MYC target genes (97, 98). Some results suggest that the phenotype and thus the clinical outcome strongly depend on the precise ratio of MAX to MYCN (100). In addition, hypoxia - in this case in particular the expression of HIF2α - is described to block differentiation and potentially also trigger dedifferentiation in neuroblastoma cells, which may contribute to a more aggressive tumor behavior (2, 101, 102). However, the role of this mechanism in *MYCN*-amplified neuroblastoma is not yet fully understood and there are also data attributing a tumor suppressor function to HIF2α in neuroblastoma (5–7). Westerlund and colleagues showed that *EPAS1* expression correlates with features of low-risk neuroblastoma (6). For clear-cell carcinomas it has been shown that HIF2α sensitizes to ferroptosis, an iron-dependent form of cell death (103). *MYCN*-amplified neuroblastoma cells are highly sensitive to ferroptosis (104), which also suggests a rather tumor-suppressive role of HIF2α in neuroblastoma. Further studies are needed to finally clarify the role of HIF2 α in neuroblastoma. The complex regulation of both signaling pathways in neuroblastoma and the resulting consequences for differentiation and metastasis, suggests a close association of the MYC and HIF signaling pathways in neuroblastomas.

Neuroblastomas occur in many of the same regions as PPGLs. New insights into the putative cellular origins of both entities came from comparative snRNA-seq analyses of human embryonal/fetal adrenal glands and neuroblastoma/PPGLs (12, 18, 105). Whereas neuroblastomas showed prominent transcriptional similarity with early normal neuroblast populations, PPGLs were transcriptionally more similar to normal cells with chromaffin cell-like morphology (**Figure 4**) (18, 105). Regulatory super-enhancer elements involved in neuroblastoma-specific chromosomal rearrangements were predominantly active in early neuroblasts of the developing adrenal glands, indicating neuroblastoma-driving events may have occurred predominantly in early neuroblast populations and not in SCP or chromaffin cell-like populations (18). The different cellular origin may also explain why PPGLs are diagnosed at older ages (median: 41.8 years; range: 5.5-83.2 years (1)) and pediatric cases are comparatively rare. Nevertheless, taking into account the low growth rate of PPGLs (volume doubling time of 5-7 years (106, 107)), it seems reasonable that tumorigenesis may be initiated during embryogenesis in at least some of these tumors (discussed later in more detail). Increased expression and stabilization of HIF2α in a specific subset of PPGLs (pseudohypoxic PPGLs) is characterized by a more immature phenotype, enhanced disease aggressiveness and onset at younger age (1, 62).

A large proportion of PPGLs are heritable due to germline pathogenic variants (PVs) in one of the described PPGL susceptibility genes (e.g. PVs in: *FH*, *SDHA*, *SDHB*, *SDHC*, *SDHD*, *SDHAF2*, *MDH2*, *VHL*, *HIF2α*, *EGLN1/2, MAX*, *TMEM127*, *NF1*, *RET*). Hereditary PPGLs are diagnosed at younger age and differ with respect to their plasma and urinary catecholamine/metanephrine profiles from sporadic PPGLs (108). Diagnosis of PPGLs largely depends on the biochemical assessment of catecholamine excess by measurement of plasma or urinary metanephrines. Due to slow growth, neglected consideration of PPGL in childhood and the immature or even non-functional catecholamine phenotype of pseudohypoxic PPGLs, the prevalence of PPGL in pediatrics may be underestimated. We recently reviewed the biochemical diagnosis of pediatric catecholamine-producing tumors and discuss in this context also the diagnosis of neuroblastoma (109).

Based on their transcriptional profile, PPGLs are divided into two main cluster groups (110, 111). Cluster 1 PPGLs bear PVs encoding two groups of genes, that either lead to a direct stabilization of HIF2α (cluster 1B, including mutations in *VHL*, *HIF2α*, *EGLN1/2*) or encode enzymes of the tricarboxylic acid (TCA) cycle (cluster 1A) that indirectly affect HIF2α stabilization (62). In contrast to the pseudohypoxic cluster 1 PPGLs, which are diagnosed earlier in life (median: 31.1 years; range: 5.5-79.3 years (1)), cluster 2 PPGLs (median: 45.2 years; range: 16.0-78-4 years) are characterized by an activation of kinase signaling pathways. The immature catecholamine phenotype of cluster 1 PPGLs is characterized by the absence of glucocorticoid-induced expression of PNMT, the rate-limiting enzyme that converts norepinephrine to epinephrine. In contrast to epinephrine-producing cluster 2 PPGLs that occur almost exclusively in the adrenal, cluster 1 PPGLs tend to metastasize more frequently and occur at intra- and extra-adrenal locations. In contrast to neuroblastomas, extra-adrenal tumor location in PPGL patients is associated with higher disease aggressiveness and shorter disease-specific survival (112). In addition, two other PPGL clusters were described based on mRNA expression analysis (111, 113). The WNT altered cluster is characterized by increased expression of genes in the WNT signaling pathway, while the cortical admixture cluster show expression of adrenal cortex markers (111).

Considering that 96% of pediatric PPGLs belong to the pseudohypoxic cluster (34% cluster 1A, 66% cluster 1B) (114), it becomes obvious that somatic mutations in cluster 1 genes likely occur during embryogenesis, while tumorigenesis of cluster 2 PPGLs may be initiated later in life. The HIF2α-mediated, pseudohypoxic PPGLs are often multifocal tumors and/or metastatic, which indicate somatic mutations even before the settlement of migratory neural crest progenitors at different locations (**Figure 4**). Moreover, paragangliomas with somatic gain-of-function mutations in *HIF2α* are associated with mosaicism and identical mutations in multiple tumors arising from postzygotic mutations at early stages of embryogenesis (115, 116). Remarkably, *HIF2α* mutations appear to be restricted to PPGLs, whereas they are mostly absent in tumor samples of other tumor entities included in The Cancer Genome Atlas (TCGA) and Genomics Evidence Neoplasia Information Exchange (GENIE) databases (117).

In PPGLs increased expression and stabilization of HIF2α is associated with a more immature/less differentiated phenotype that is accompanied by increased disease aggressiveness (1, 118). PPGLs with enhanced expression/stabilization of HIF2α do not express PNMT, whereas HIF2α expression in neuroblastomas marks a subpopulation of immature neural crest-like cells (2, 119). The induced expression of HIF2α target genes and the associated pseudohypoxic environment may provide cells of cluster 1 PPGLs with a selection advantage that favors tumorigenesis in these cells. This hypothesis is supported by patients with PPGL that occurred as a complication of cyanotic congenital heart disease (CCHD). These patients suffer from chronic hypoxia and frequently carry somatic PVs in *HIF2α* (120–122). PVs in *HIF2α* or already an increased HIF2α expression during neural crest may provide a survival benefit for neural crest derived cells. The organ of Zuckerkandl, the largest source of extra-adrenal chromaffin cells in mammals, disappears postnatally by a glucocorticoid-mediated mechanism of autophagy (123). An impairment of this mechanism during embryogenesis or immediately after birth may initiate premature paraganglioma formation. Unlike in neuroblastoma, where spontaneous but also drug-induced transitions from different stages of differentiation are observed (97), in the case of PPGLs this hypothesis would tend to suggest that the differentiation status is largely determined by the cellular origin and the underlying mutation (124). The slow growth and comparatively low aggressiveness of PPGLs, indicating a comparable low cellular flexibility, would potentially further support this hypothesis.

In hereditary PPGL patients, multifocal and bilateral tumors are associated with an earlier onset of the disease than in patients with solitary PPGLs (108). This might be explained by the initiation (second hit) of these tumors from a single tumor stem cell already during neural crest cell migration (Knudson two-hit hypothesis (125)). This would imply that in some patients with germline PVs the second chromosomal hit already occurs during embryogenesis, before the neural crest cells migrate to their paraganglial or adrenal localization.

Germline PVs in *MAX* are associated with bilateral and multifocal PPGLs (67%) (126, 127) and the occurrence of both neuroblastomas and PPGLs (128, 129), which highlights the importance of the MYC/MAX convergence point in these catecholamine-producing neoplasms. Furthermore, it suggests that the chromosomal second hit can occur early enough in embryogenesis to allow differentiation of the migrating neural crest cells into both, neuroblasts and chromaffin cells. Although tumors with *MAX* loss-of-function mutations classically belong to cluster 2 PPGLs, data on catecholamines suggest a biochemical phenotype intermediate between the established epinephrine-producing phenotype of cluster 2 PPGLs and a norepinephrine-producing phenotype of cluster 1 PPGL (22, 118, 127). PPGLs with *MAX* mutations belong to a specific molecular subgroup defined as cortical admixture subtype, which overexpress both PPGL markers and adrenal cortex markers (111). The intermediate phenotype of *MAX*-mutated PPGLs suggests that a fully functional MYC/MAX complex is required to facilitate differentiation. Whether c-Myc and MYCN assign different roles by the manifestation of the different phenotypic feature remain unclear. However, it would be reasonable to assume that they exert different functions, given their role in neural crest cell differentiation and migration.

A better understanding of the underlying cellular and molecular mechanisms also opens up for new therapeutic approaches to treat these catecholamine-producing neoplasms of the neural crest. In addition to the involvement of HIF2α in tumorigenesis of PPGLs, we and others also demonstrated the involvement of HIF2α in PPGL metastasis (1, 62, 130, 131), as reflected by the increased metastasis rate of pseudohypoxic cluster 1 PPGLs compared with cluster 2 PPGLs (1). Through the development of selective small-molecule inhibitors targeting HIF2α/ARNT dimerization, potentially suitable drugs are available, which are of particular interest for the more aggressive pseudohypoxic PPGLs and neuroblastomas. Nevertheless, some preclinical PPGL and neuroblastoma models show a lack of efficiency of these inhibitors (1, 63, 132), which is also in line with some clinical data from other HIF2α-dependent tumor entities that show resistance towards the available HIF2α inhibitors (133, 134). This partial lack of efficiency in HIF2α-dependent tumors may provide preliminary evidence that mechanisms independent of ARNT/HIF2α dimerization, for instance through interactions with the MYC/MAX complex (27, 34, 39), are involved in tumorigenesis and metastasis of these tumors. The ARNT-independent mechanisms of HIF2α may offer alternative therapeutic approaches for some patients who exhibit resistance to HIF2α inhibitors or may even be more efficient.

Direct therapeutic targeting of MYC/MYCN has been a challenge for decades considering its “undrugability” protein structure, but a least some small molecules are available that inhibit dimerization of MYC/MAX (135). However, this approach has shown only limited effectiveness (136). This may indicate other factors that influence the complex and thus MYC-target gene expression. For example, HIF2a expression in neuroblastomas is also associated with increased aggressiveness and a more undifferentiated phenotype. Similar to observations in PPGLs, ARNT-dependent inhibition of HIF2α by PT2385 (HIF2α inhibitor) is not sufficient to regulate HIF2α downstream target genes in neuroblastoma (63), which also indicates a ARNT-independent mechanisms of HIF2α in neuroblastoma (see discussion above). This further underlines the potential importance of the HIF-MYC/MAX interaction in these tumors.

## 6 Conclusion

Emerging single-cell methods have provide further insight in neural crest-derived cell linages, particularly in the adrenal gland, while epigenetic and transcriptomic data have increased our knowledge of tumorigenesis and progression of adrenal neoplasms. A tight regulation of HIF and MYC signaling pathways during neural crest development appears to be essential, among other things, for the complete and correct formation of the adrenal medulla and the sympathetic and parasympathetic ganglia. Dysregulation in either pathway may cause the emergence of neuroblastomas or pheochromocytomas, associated with an undifferentiated and more aggressive phenotype. Especially in pheochromocytomas/paragangliomas, increased expression and stabilization of HIF2α causes these tumors to appear at a younger age. An improved understanding of the cellular origin and pathogenesis of these pediatric adrenal neoplasms will contribute to a more efficient subtype classification and allow more precise and effective treatment of these young patients; in particular, the HIF-MYC/MAX interaction should be further considered.

## Author contributions

NB carefully reviewed the literature and wrote the first version of the manuscript. FW and GE provided conceptual input, contributed to the editing, and revised the manuscript. All authors contributed to the article and approved the submitted version.
